# Prevalence of Asthma and Allergic Rhinitis among Adults in Yaounde, Cameroon

**DOI:** 10.1371/journal.pone.0123099

**Published:** 2015-04-08

**Authors:** Eric Walter Pefura-Yone, André Pascal Kengne, Adamou Dodo Balkissou, Julie Raïcha Boulleys-Nana, Nelly Rachel Efe-de-Melingui, Patricia Ingrid Ndjeutcheu-Moualeu, Charles Lebon Mbele-Onana, Elvira Christelle Kenmegne-Noumsi, Barbara Linda Kolontchang-Yomi, Boris Judicaël Theubo-Kamgang, Emilienne Régine Ebouki, Chrystelle Karen Djuikam-Kamga, Christiane Gaelle Magne-Fotso, Francine Amougou, Liliane Mboumtou, Martine Ngo-Yonga, Elsie Linda Petchou-Talla, Emmanuel Afane-Ze, Christopher Kuaban, Research Group for Respiratory Disease in Cameroon (RGRDC)

**Affiliations:** 1 Department of Internal Medicine and Subspecialties, Faculty of Medicine and Biomedical Sciences, University of Yaounde I, Yaounde, Cameroon; 2 Pneumology service, Yaounde Jamot Hospital, Yaounde, Cameroon; 3 South African Medical Research Council, Cape Town, South Africa; 4 University of Cape Town, Cape Town, South Africa; 5 Biyem’assi District Hospital, Yaounde, Cameroon; 6 Institut Supérieur de Technologie Médicale, Yaounde, Cameroon; 7 Faculty of Heath Sciences, University of Bamenda, Bamenda, Cameroon; Université Libre de Bruxelles, BELGIUM

## Abstract

**Background:**

Population-based estimates of asthma and allergic rhinitis in sub-Saharan African adults are lacking. We assessed the prevalence and determinants of asthma and allergic rhinitis in urban adult Cameroonians.

**Methods:**

A community-based survey was conducted from December 2013 to April 2014 among adults aged 19 years and above (N = 2,304, 57.3% women), selected through multilevel stratified random sampling across all districts of Yaounde (Capital city). Internationally validated questionnaires were used to investigate the presence of allergic diseases. Logistic regressions were employed to investigate the determinants of allergic conditions.

**Results:**

Prevalence rates were 2.7% (95% CI: 2.1-3.4) for asthma-ever, 6.9% (5.9-7.9) for lifetime wheezing, 2.9% (92.2-3.6) for current wheezing and 11.4% (10.1-12.7) for self-reported lifetime allergic rhinitis; while 240 (10.4%) participants reported current symptoms of allergic rhinitis, and 125 (5.4%) had allergic rhino-conjunctivitis. The prevalence of current asthma medication use and self-reported asthma attack was 0.8 (0.4-1.2) and 1 (0.6-1.4) respectively. Multivariable adjusted determinants of current wheezing were signs of atopic eczema [2.91 (1.09-7.74)] and signs of allergic rhinitis [3.24 (1.83-5.71)]. Age group 31-40 years [0.27(0.09-0.78), p = 0.016] was an independent protective factor for wheezing. Determinants of current rhinitis symptoms were active smoking [2.20 (1.37-3.54), p<0.001], signs of atopic eczema [2.84 (1.48-5.46)] and current wheezing [3.02 (1.70-5.39)].

**Conclusion:**

Prevalence rates for asthma and allergic rhinitis among adults in this population were at the lower tails of those reported in other regions of the world. Beside the classical interrelation between allergic diseases found in this study, active smoking was an independent determinant of allergic rhinitis symptoms. Nationwide surveys are needed to investigate regional variations.

## Introduction

Allergic respiratory conditions are a major public health challenge worldwide. According to the World Health Organization (WHO) estimates, about 300 million people around the world suffer from asthma and about 250,000 people die from the disease each year [1]. Likewise about 12% to 30% of the population across regions suffer from allergic rhinitis [2]. Although allergic rhinitis is not a fatal condition, it is associated with impaired quality of life, absenteeism from work and substantial financial costs [3].

Reports from epidemiological studies suggest increases in the prevalence of respiratory allergic diseases in the last decades [4,5]. There has also been improvement in the knowledge regarding the risk factors of these diseases which include among others host related factors such as genetic predisposition, smoking, obesity, hormonal changes, and environmental factors comprising exposition to aeroallergens, improved hygiene, air pollution and lifestyle changes with adoption of western diet [6].

In sub-Saharan Africa, little is known on the prevalence of asthma and allergic rhinitis in adults [7,8]. Furthermore, the few studies published over ten years ago indicate prevalence figures among adults ranging from 24.4% to 30% for current allergic rhinitis [9,10], and 2% to 12% for current asthma symptoms (wheezing) [11,12,13]. However, the prevalence of self-reported asthma varies from one African country to another. The prevalence of self-reported asthma in Tanzania, Nigeria and South Africa was 3.3%, 2% and 3.8% respectively [12,13,14]. The only available prevalence study of asthma in adults in Cameroonians was conducted during 1996–1997, and reported a prevalence of 1.7% for wheezing in the last 12 months [13]. We are not aware of existing prevalence studies on allergic rhinitis in Cameroon.

The aim of this study was to assess the prevalence and determinants of asthma and allergic rhinitis in urban adult Cameroonians.

## Material and Methods

### Study type and population

This cross-sectional study was conducted in the seven districts of Yaounde, the Capital City of Cameroon, from December 2013 to April 2014 (five months). Yaounde is situated at about 700–800 m above the sea and had a total population of 2,440,462 inhabitants in 2011 [15]. Consenting adults aged 19 years and above were considered for inclusion in the study[16]. People with hearing and speech problems were excluded.

### Procedures

#### Sampling

A multilevel stratified random procedure was used to select participants for inclusion in this study. The first level consisted of randomly selecting 16 enumeration areas (EA) from a total of 2000 in Yaounde, based on a ratio of 2–3 EA per district. EA delineation was based on the last general population census conducted in Cameroon in 2005[17]. At the second level one household in two was selected using a systematic sampling (sampling step = 2). The number of households per EA ranged from 70 to 110. The first household and itinerary followed were based on those of the national vaccination campaigns (NVC). At the third level all adults aged 19 years and above in selected households formed the primary statistical units for data collection.

### Data collection

All consenting participants were included in the study. Data collection used a questionnaire derived from those of the International Study of Asthma and Allergies in Childhood (ISAAC)[18] and the European Community Respiratory Health Survey II (ECRHS II)[19]. Questionnaires were completed by trained field workers who were all final year undergraduate medial students. Socio-demographic details included sex, age, marital status, region of origin and ethnic group, the highest education level achieved and the number of individuals per bedroom in the household. Tobacco smoking was assessed and participants ranked as current smokers (participants who reported having smoked at least one cigarette per day for at least one year, or having smoked at least 20 cigarettes in their lifetime and was still smoking), ex-smokers (participants who declared having stopped smoking for at least six months) and non-smokers[20].

Data collected on asthma and asthma symptoms included: asthma diagnosed by a health professional, asthma crisis in the last twelve months, chest wheezing at any time, effort related wheezing, severity of wheezing in the last twelve months (number of wheezing, number of times awaken by wheezing, wheezing episodes with inability to speak out one or two words consecutively)[18], awakening due to tightness, awakening due to attack of breathless, awakening due to cough, night-time dry cough in the absence of flu and chest infection, current medication intake for asthma. Data collected for the diagnosis of allergic rhinitis and rhino-conjunctivitis included: signs of rhinitis (sneezing, rhinorrhoea, nasal obstruction) in the absence of flu and cold, tears and itching during rhinitis, impairment of daily life activities due to allergic rhinitis. The presence of atopic eczema was based on the existence of characteristic symptoms which are « skin eruptions (reddish papule, swollen spot…), which are itching, and appear and disappear intermittently over a period of at least six months ». Height and weight were measured and body mass index (BMI) derived as weight (kg)/height/height (m^2^), and BMI>30kg/ m^2^ used to define obesity[21].

### Operational definitions [18,19]

Lifetime asthma diagnosis (asthma-ever) was based on self-reported diagnosis of asthma by a health professional during the lifetime of the participant. Current symptoms of asthma were those experienced in the last 12 months. Rhinitis was defined by sneezing, nasal blockage or rhinorrhoea at the time the subject did not have cold or flu. Current symptoms of allergic rhinitis were those experienced in the last 12 months. Lifetime allergic rhinitis was based on self-reported allergic rhinitis during the lifetime of the participant.

### Ethics statement

The study was approved by the Ethics Committee of the Faculty of Medicine and Pharmaceutical Sciences of the Douala University, Cameroon. The study was further approved by the health authorities of the Centre Region of Cameroon, and signed informed consent obtained from all included participants.

### Statistical Analysis

We have used sample size calculation module of Epi-info software to derive the sample size. The inflation factor was based on the recruitment of 156 subjects in each enumeration era and a hypothetical intraclass correlation coefficient (σ) of 0.006 [22]. The design effect or inflation factor is computed as 1+σ(m-1), where m = 156 is a size of clusters. Thus, the design effect is 2 for this study. Based on an estimated population of 1.4 million inhabitants, considering a prevalence of 1.5% for current wheezing, for a precision of 1% and a 95% confidence interval, the required sample size for the current study was 605 individuals. Considering an inflation factor of two for the effect of stratification and a non-response rate of 10%, the minimal required number of participants was 1331.

Data were analysed with the use of IBM-SPSS v.20 for Windows (SPSS Inc., Chicago, IL). Qualitative data are presented as counts and proportions, and quantitative variables as mean and standard deviation (SD) or median and 25th-75th percentiles. Chi square test and Fisher exact test were used to compare proportions. Age-standardized prevalence was calculated using the Cameroon National population’s age structure in 2010 as the standard population [23], and direct standardization methods [24]. Logistic regressions models were employed to investigate the determinants of current wheezing and current allergic rhinitis. Potential determinants were first tested in univariable analysis and significant determinants (based on a threshold p<0.10) were further tested in multivariable models. A p-value<0.05 was used to characterise statistically significant results.

## Results

### General characteristics of the study population

A total of 2,475 participants were invited to take part in the study, of whom 194 declined (response rate 93.4%), and survey forms were incomplete for seven other participants. Therefore 2,304 participants were included in the final analytic sample. The characteristics of this sample are presented in Table 1. In all 1321 (57.3%) were women, mean age (SD) was 34.9 (13.5) years, and 32% had university education, while 8.4% were current smokers and 5.6% were ex-smokers.

**Table 1:** Characteristics of study population.

Characteristics	Overall	Women	Men	p-value
N	2304 (100%)	1321 (57.3%)	983 (42.7%)	
Age, years				
Range	19–90	19–87	19–90	
Mean (standard deviation)	34.9 (13.5)	35.1 (13.3)	34.6 (13.7)	0.412
Median (25^th^-75^th^ percentiles)	30 (24–42)	31 (25–43)	30 (24–42)	
Level of education				
Range	0–22	0–22	0–20	
Mean (standard deviation)	11.3 (4.2)	10.6 (4.3)	12.2 (3.9)	<0.001
Median(25^th^-75^th^ percentiles)	12 (8–15)	10 (7–14)	12 (10–15)	
≤ Primary school, n (%)	423/2297 (18.4)	305 (23.1)	118 (12)	<0.001
Secondary school, n (%)	1136/2297 (49.5)	657 (49.9)	479 (48.9)	
Higher education, n (%)	738/2297 (32.1)	355 (27.0)	383 (39.1)	
Ethnic group				<0.001
Bantou, n (%)	1191/2291 (52)	704 (53.7)	487 (49.6)	
Semi-Bantou, n (%)	970/2291 (42.3)	556 (42.4)	414 (42.2)	
Others, n (%)	130/2291 (5.7)	50 (3.8)	80 (8.2)	
Marital status				0.014
Living in couple, n (%)	802/2274 (35.3)	488 (37.4)	314 (32.4)	
Alone, n (%)	1472/2274 (64.7)	816 (62.6)	656 (67.6)	
Number/bedrooms in household				
Range	0.3–11	0.3–11.0	0.3–9.0	
Mean (standard deviation)	2.4 (1.4)	2.6 (1.4)	2.2 (1.3)	<0.001
Median (25^th^-75^th^ percentiles)	2.1 (1.5–3)	2.2 (1.7–3.0)	2.0 (1.0–2.7)	
Tobacco smoking				<0.001
Smokers, n (%)	194/2298 (8.4)	36 (2.7)	158 (16.1)	
Ex-smokers, n (%)	129/2298 (5.6)	25 (1.9)	104 (10.6)	
Non-smokers, n (%)	1975/2298 *85.9)	1257 (95.4)	718 (73.3)	
BMI, kg/m^2^				
Range	14.2–60.4	14.2–60.4	15.2–49.9	
Mean (standard deviation)	26.5 (5.4)	27.4 (5.9)	25.1 (4.3)	<0.001
Median(25^th^-75^th^ percentiles)	25.3 (22.6–29.4)	26.6 (23.1–30.8)	24.4 (22.2–27.6)	
≥ 30, n (%)	502/2280 (22)	379 (29.0)	123 (12.7)	<0.001

BMI, body mass index.

### Prevalence of asthma, symptoms of asthma and allergic rhinitis

The prevalence of asthma-ever was 2.7% (95% confidence interval: 2.1–3.4%). A total of 158 participants had had at least an episode of wheezing in their lifetime, giving a prevalence of 6.9% (5.9–7.9) for wheeze-ever. The prevalence of current wheezing was 2.9% (2.2–3.6). The crude and age-standardised prevalence of symptoms of asthma is found in Table 2. Night-time awakening by coughing attack was the most frequent symptom with a prevalence of 15.3% (13.8–16.8), followed by nocturnal dry cough [10.6% (9.3–11.9)]. The prevalence of self-reported lifetime allergic rhinitis was 11.4% (10.1–12.7). Current allergic rhinitis symptoms were reported by 240 participants (10.4%) and 125 (5.4%) had allergic rhino-conjunctivitis symptoms. With the exception of asthma-ever (p = 0.036) and current asthma (p = 0.017), prevalence rates were mostly similar in men and women; furthermore, age-standardisation had meaningless effect on the overall prevalence rates (Table 2).

**Table 2:** Prevalence of asthma, asthma symptoms and rhinitis.

Symptoms	Number	Crude prevalence, % (95% CI)	p gender	Age Standardized prevalence, Overall, % (95% CI)	Age Standardized prevalence for men, % (95% CI)	Age Standardized prevalence for women, % (95% CI)
**Asthma**						
Asthma ever	65	2.7 (2.1–3.4)	0.036	2.7 (2.0–3.4)	1.6 (0.8–2.4)	3.5 (2.4–4.6)
Current attack of asthma	24	1 (0.6–1.4)	0.017	1.2 (0.7–1.7)	0.7 (0.2–1.2)	1.6 (0.9–2.3)
Current asthma medications use	19	0.8 (0.4–1.2)	0.225	0.8 (0.4–1.2)	0.4 (0.0–0.8)	1.2 (0.6–1.8)
Wheezing ever	158	6.9 (5.9–7.9)	0.325	6.9 (5.8–8.0)	6.3 (4.7–7.9)	7.4 (5.9–8.9)
Current wheezing	66	2.9 (2.2–3.6)	0.675	2.9 (2.2–3.6)	2.6 (1.6–3.6)	3.1 (2.1–4.1)
Current wheezing at effort	62	2.7 (2.0–3.4)	0.611	2.6 (2.0–3.2)	2.3 (1.3–3.3)	2.7 (1.8–3.6)
Current awakening with tightness	217	9.4 (8.2–10.6)	0.244	9.3 (8.0–10.6)	8.5 (6.6–10.4)	10.1 (8.4–11.8)
Current attack of breathless	137	5.9 (4.9–6.9)	0.076	6.2 (5.2–7.2)	5.1 (3.6–6.6)	6.8 (5.4–8.2)
Current attack cough	352	15.3 (13.8–16.8)	0.138	15.2 (13.6–16.8)	13.4 (11.1–15.7)	16.4 (14.2–18.6)
Current nocturnal cough without cold	245	10.6 (9.3–11.9)	0.685	10.7 (9.3–12.1)	11.0 (8.9–13.1)	10.6 (8.8–12.4)
**Rhinitis**						
Self-reported allergic rhinitis	262	11.4 (10.1–12.7)	0.247	11.2 (9.8–12.6)	12.1 (9.9–14.3)	10.4 (8.7–12.1)
Rhinitis symptoms ever	342	14.8 (13.4–16.2)	0.389	14.6 (13.0–16.2)	13.5 (11.2–15.8)	15.3 (13.2–17.4)
Current symptoms of rhinitis	240	10.4 (9.2–11.6)	0.591	10.3 (9.0–11.6)	9.4 (7.5–11.3)	10.8 (9.0–12.6)
Current rhino-conjunctivitis	125	5.4 (4.5–6.3)	0.377	5.3 (4.3–6.3)	4.7 (3.3–6.1)	5.8 (4.5–7.1)

CI, confidence interval.

### Severity of the wheezing and allergic rhinitis

Of the 66 participants with current wheezing, 10.6% had more than 12 episodes of wheezing in the last 12 months; 12.1% had more than one episode of sleep disturbances per week due to wheezing, while 43.9% had had severe attacks with inability to say one or two words during wheezing (Table 3). Of the 306 participants with symptoms of allergic rhinitis who had provided information on the impact of symptoms on daily life activities, 11 (3.6%) had severe impairment, 14 (4.6%) had moderate impairment, 55 (18%) had mild impairment while 226 (73.9%) had no impairment.

**Table 3:** Severity of current wheezing.

Severity indices	Prevalence, %
Number of wheezing episodes in 12 months	
≤3	80.3
4–12	9.1
> 12	10.6
Sleep disturbed by wheezing	
Never	57.6
< 1/week	30.3
≥ 1/week	12.1
Limitation of speech to one or two words during wheezing	
Yes	43.9
No	56.1

### Determinants of asthma and allergic rhinitis

**Fig 1:**
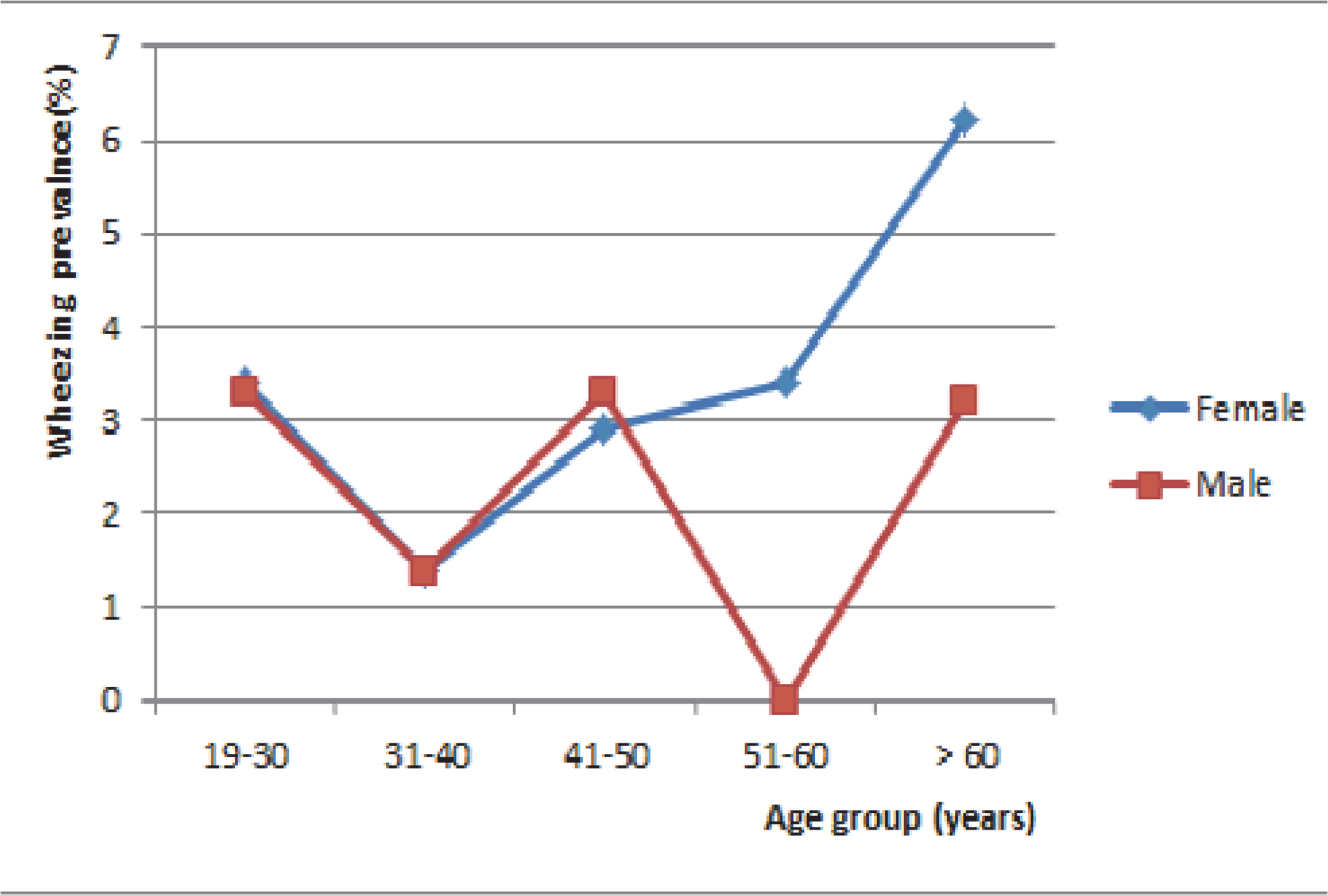
Prevalence of current wheezing by age group and gender.

In univariable analysis, current wheezing was less frequent in the age group 31–40 years relative to other age groups. The prevalence of current wheezing by sex and age groups is shown in Fig 1. Current wheezing was higher in women aged 50 years and above, relative to their male counterparts (p = 0.128). Current wheezing was found in 7.5% of participants with rhinitis and in 2.3% of those without rhinitis (p<0.001). The prevalence of current rhinitis was 27.3% among subjects with current wheezing and 9.9% in those without current wheezing (p<0.001). Six teen (25.4%) participants with asthma ever had current rhinitis and 10% of participants without asthma ever had current rhinitis (p<0.001). Likewise, the prevalence of wheezing was higher in participants with signs of eczema compared to those without (9.4% vs. 2.7%, p<0.001). Allergic rhinitis was more frequent in participants with university education, in smokers and participants with wheezing or symptoms of atopic eczema (Table 4).

**Table 4:** Prevalence and odds ratio of current wheezing and current allergic rhinitis in selected group.

Characteristics		Current wheezing			Asthma ever			Current rhinitis symptoms			Current rhino-conjunctivitis symptoms	
	%	COR (95% CI)	p	%	COR (95% CI)	p	%	COR (95% CI)	p	%	COR (95% CI)	p
**Sex**												
Female	3	1.15 (0.70–1.90)	0.589	3.7	2.67(1.46–4.86)	0.001	10.7	1.09 (0.83–1.43)	0.504	5.8	1.21(0.83–1.75)	0.322
Male	2.6	1		1.4	1		10	1		4.9	1	
**Age group, years**												
19–30	3.3	0.68 (0.30–1.54)	0.297	3.1	1.1250.39–3.18)	0.839	11.3	1.40 (0.76–2.61)	0.282	6.5	1.37(0.62–3.02)	0.443
31–40	1.4	0.29 (0.10–0.81)	0.018	2.4	0.86(0.27–2.71)	0.798	9.8	1.20 (0.62–2.31)	0.597	3.6	0.73(0.30–1.79)	0.492
41–50	3	0.61 (0.22–1.67)	0.336	3	1.09(0.33–3.60)	0.888	8.7	1.05 (0.51–2.15)	0.891	3	0.61(0.22–1.67)	0.336
51–60	2	0.41 (0.12–1.42)	0.408	1	0.36(0.07–2.00)	0.243	10.7	1.32 (0.63–2.78)	0.465	7.7	1.62(0.64–4.09)	0.305
> 60	4.9	1		2.8	1		8.3	1		4.9	1	
**Level of education**												
< secondary	3.1	0.94 (0.48–1.87)	0.867	2.6	0.79(0.39–1.64)	0.533	9.2	1		5.9	1	
secondary	2.6	0.78 (0.45–1.35)	0.373	2.5	0.75(0.43–1.31)	0.312	8.8	0.95 (0.65–1.40)	0.797	4.4	0.86(0.53–1.42)	0.564
Higher education	3.3	1		3.3	1		13.7	1.56 (1.06–2.31)	0.025	6.8	1.58(1.05–2.36)	0.027
**Ethnic group**												
Bantou	3.2	2.11 (0.50–8.85)	0.308	3	1.32(0.40–4.35)	0.648	11	1.89 (0.90–3.94)	0.092	5.8	1.27(0.54–2.99)	0.582
Semi-Bantou	2.7	1.76 (0.41–7.52)	0.444	2.4	0.97(0.29–3.29)	0.964	10.4	1.77 (0.84–3.73)	0.132	5.2	1.12(0.47–2.67)	0.793
Peul/Sudanese	1.5	1		3	1		6.2	1		4.6	1	
**Marital status**												
Living in couple	2.4	1		1.9	1		9	1		4.1	1	
Alone	3.2	1.36 (0.79–2.33)	0.263	3.3	1.77(0.98–3.18)	0.054	11.3	1.30 (0.97–1.74)	0.079	6.2	1.54(1.02–2.31)	0.038
**Number/chamber in household (n = 2232)**												
≤ 2	3.2	1.29 (0.78–2.13)	0.321	3	1.27(0.76–2.12)	0.356	11.2	1.201 (0.92–1.58)	0.174	5.6	1.06(0.74–1.53)	0.749
> 2	2.5	1		2.4	1		9.5	1		5.3	1	
**Tobacco smoking (n = 2298)**												
smokers	3.1	1.09 (0.39–3.07)	0.861	1.5	0.51(0.16–1.64)	0.259	19.4	2.21 (1.39–3.50)	0.001	8.2	1.70(0.98–2.95)	0.058
Ex-smokers	3.1	1.09 (0.47–2.57)	0.837	0.8	0.25(0.04–1.85)	0.176	10.8	1.11 (0.69–1.80)	0.656	7.8	1.59(0.81–3.13)	0.178
Non-smokers	2.8	1		3	1		9.8	1		5	1	
**History of pulmonary TB**												
Yes	2.2	0.77 (0.10–5.65)	0.794	4.4	1.68(0.39–7.08)	0.477	11.1	1.08 (0.42–2.76)	0.878	2.2	0.39(0.05–2.84)	0.388
No	2.9	1		2.7	1		10.4	1		5.5	1	
**BMI, kg/m** ^**2**^ **(n = 2280)**												
≥ 30	3	0.96 (0.53–1.72)	0.88	3.4	1.35(0.77–2.38)	0.300	10.6	1.04 (0.75–1.43)	0.834	4.4	0.77(0.48–1.23)	0.276
< 30	2.9	1		2.5	1		10.2	1		5.6	1	
**Current eczema symptoms**												
Yes	9.4	3.74 (1.44–9.72)	0.016	5.7	2.19(0.67–7.22)	0.175	26.4	3.22 (1.72–6.02)	<0.001	18.9	4.32(2.12–8.81)	<0.001
No	2.7	1		2.7	1		10	1		5.1	1	
**Current rhinitis**							/	/	/	/	/	/
Yes	7.5	3.41 (1.95–5.96)	<0.001	6.7	3.07(1.71–5.50)	<0.001	/	/	/	./	/	/
No	2.3	1		2.3								
**Current wheezing**												
Yes	/	/	/	30.3	22.19(12.11–40.67)	<0.001	27.3	3.41 (1.95–5.96)	<0.001	20	4.70(2.49–8.89)	<0.001
No	/	/	/	1.9	1		9.9	1		5	1	
**Asthma ever**												
Yes	31.7	22.19(12.11–40.66)	<0.001	/	/	/	25.4	3.41 (1.95–5.96)	<0.001	15.9	3.46(1.71–6.97)	<0.001
No	2.1	1		/	/	/	10			5.2	1	

CI, confidence interval; CRO, crude odds ratio; BMI, body mass index; TB, tuberculosis.

In multivariable regression analyses (Table 5), significant determinants of current wheezing were signs of atopic eczema [adjusted odd ratio (95% CI): 2.91 (1.09–7.74), p = 0.033] and symptoms of allergic rhinitis [3.24 (1.83–5.71), p < 0.001]. Age group 31–40 years [0.27 (0.09–0.78), p = 0.016] was an independent protective factor for wheezing. Female gender [2.76 (1.51–5.05), p = 0.001] and current rhinitis symptoms [2.99 (1.66–5.39), p<0.001)] were the independent determinants of asthma ever. Determinants of rhinitis were current smoking [2.20 (1.37–3.54), p <0.001], signs of atopic eczema [2.84 (1.48–5.46), p = 0.002] and current wheezing [3.02 (1.70–5.39), p < 0.001]. Finally, determinants of rhino-conjunctivitis were current wheezing [4.06 (2.11–7.81), p<0.001] and atopic eczema [3.53 (1.67–7.47) p = 0.001].

**Table 5:** Multivariate analysis of factors associated to current wheezing and current rhinitis symptoms in Yaounde, Cameroon.

CURRENT WHEEZING		
Factors	Adjusted OR (95% CI)	p
Age group, years		
19–30	0.64(0.28–1.47)	0.290
31–40	0.27(0.09–0.78)	0.016
41–50	0.61(0.22–1.68)	0.340
51–60	0.40(0.11–1.39)	0.149
> 60	1	
Eczema symptoms		
Yes	2.91(1.09–7.74)	0.033
No	1	
Rhinitis		
Yes	3.24(1.83–5.71)	<0.001
No	1	
**ASTHMA EVER**		
Factors		
Sex		
Female	2.76(1.51–5.05)	0.001
Male	1	
Age group, years		
19–30	0.89(0.30–2.61)	0.829
31–40	0.81(0.25–2.57)	0.716
41–50	1.17(0.34–3.91)	0.802
51–60	0.37(0.07–2.10)	0.263
> 60	1	
Marital status		
Living in couple	1	
Living alone	1.79(0.94–3.40)	0.078
Current rhinitis symptoms		
Yes	2.99(1.66–5.39)	<0.001
No	1	
**CURRENT RHINITIS SYMPTOMS**		
Factors	Adjusted OR (95% CI)	p
Level of education		
< secondary	1	
Secondary	0.92(0.62–1.37)	0.680
Higher education	1.49(1.00–2.23)	0.051
Ethnic group		
Bantou	1.72(0.81–3.62)	0.156
Semi-Bantou	1.69(0.80–3.58)	0.170
Peul/Sudanese	1	
Marital status		
Alone	1,19(0.88–1.62)	0.250
Living in couple	1	
Tobacco smoking		
Smokers	2.20(1.37–3.54)	0.001
Ex-smokers	1.12(0.69–1.83)	0.646
Non-smokers	1	
Eczema symptoms		
Yes	2.84(1.48–5.46)	0.002
No	1	
Wheezing		
Yes	3.02(1.70–5.39)	<0.001
No	1	
**CURRENT RHINO-CONJUNCTIVITIS**		
Factors		
Level of education		
< secondary	1	
Secondary	0.70(0.43–1.16)	0.166
Higher education	1.07(0.64–1.79)	0.793
Marital status		
Alone	1.45(0.95–2.22)	0.082
Living in couple	1	
Tobacco smoking		
Smokers	1.64(0.93–2.88)	0.087
Ex-smokers	1.58(0.79–3.19)	0.193
Non-smokers	1	
Eczema symptoms		
Yes	3.53(1.67–7.47)	0.001
No	1	
Wheezing		
Yes	4.06(2.11–7.82)	<0.001
No	1	

OR, odds ratio.

## Discussion

The key finding from this first study on the prevalence of allergic rhinitis and asthma among adults in urban Cameroon are the following: 1) about three percent of this population had current wheezing or a self-reported lifetime diagnosis of asthma; 2) about one in ten participants had symptoms of allergic rhinitis while one in twenty participants had symptoms of rhino-conjunctivitis; 3) Finally, other allergic conditions were independently associated with asthma and allergic rhinitis.

The prevalence of asthma and symptoms varies across regions [8,25]. In general, the prevalence of self-reported asthma is lower than that for wheezing, the chief symptoms of asthma used in epidemiological studies. The prevalence of asthma and symptoms is higher in northern Europe, USA, Australia and New Zealand [25]. The prevalence of current wheezing is as higher as 32% in these countries and the prevalence of self-reported current asthma as higher as 13% [25,26]. Contrariwise, prevalence rates are lower in Southern Europe (Italy and Spain) where prevalence rates for wheezing and self-reported asthma range from 4 to 13.9% and from 2 to 6.6% respectively [25,27]. Significant variations in the prevalence of asthma and symptoms are strong indicators of the need for locally relevant studies. In our study conducted in a major sub-Saharan African city, the prevalence of current wheezing was 2.9%. This prevalence is much lower than those reported in most European countries, but close to rates reported in most Asian countries [25,28,29].

Very few studies are available on the epidemiology of asthma in sub-Saharan African countries, particularly in adults. The prevalence of self-reported asthma-ever in our study is close to that reported in Tanzania (3.3%) [13], Nigeria (2%) [12] and South Africa (3.8%) [14]. Wheezing in the last 12 months was reported by 2.9% of our participants. Rates reported from other African countries ranged from 1.7% in Ethiopia, 3% in Tanzania, 9% in Nigeria to 16.3% in South Africa [11,13,14,30]. The prevalence of lifelong wheezing was 6.9% in our participants. The prevalence of asthma in Yaounde was at the lower tail of figures reported across other regions in the world. In a systematic review conducted by Adeloye et al comprising prevalence studies on Asthma from Africa published between 1990 and 2013, the estimated overall prevalence of asthma (current wheezing) in Africa was 13.8% (95%CI: 6.2–21.4) in people aged 15 to 45 years [8]. This figure is higher than the one reported in our study, and could be explained at least in part by the fact that the review by Adeyole et al included more adolescents, with the average age of 18.4 years.

The main suggested reasons to explain these regional disparities in the prevalence of asthma include the exposure to environmental factor (climatic variations, exposure to aeroallergens, air pollution, exposure to micro-organisms), socio-economic factors, and lifestyles [31,32]. Mites’ sensitisation is an important and powerful risk factor of asthma[33]. Sensitisation to mites is frequent in people with asthma in Yaounde and in other African countries [34,35,36]. The level of this sensitisation is comparable to that reported in most regions of the world [34,35,36,37] and may therefore not explain the low prevalence of asthma in most African countries. The low prevalence of smoking found in our study and which has also been reported in other African countries [12,38], could partially explain the low prevalence of asthma in Yaounde and across Africa. Other risk factors for asthma such as obesity and low socio-economic status [39] were not associated with asthma or wheezing in this study. Complex interactions between environmental factors and host related factors (genetic, comorbidities, lifestyle) further account for some of the variations in asthma prevalence around the world.

More than 40% of our participants, who had had wheezing in the last 12 months, had severe wheezing. Of those patients with severe wheezing, less than 1/3rd took asthma medications in the last 12 months. These results suggest a potentially fatal discrepancy in the management of people with asthma in this setting. As classically reported, asthma symptoms were more frequent in participants with other allergic conditions [40]. Indeed, participants with current wheezing were three times more likely to have rhinitis and atopic eczema.

Symptoms of current allergic rhinitis and rhino-conjunctivitis were present in one in ten and one in twenty participants respectively. Symptoms of allergic rhinitis were less frequent in our study than in reports from Asia where prevalence ranged from 23.6% to 38% [2,41] and Nigeria where it was 29.6% [9]. Self-reported allergic rhinitis in our study (11.4%) was within the range of reports from other parts of the world: 13.1% in Europe (ranging from 11.1% in Italy to 16.2% in Germany), 14% in USA, 7% in Latin America and 9% in Asia Pacific [42,43]. In addition to the classical association between allergic rhinitis and other allergic conditions, smoking and higher education were more frequent in our participants with allergic rhinitis. The high prevalence of allergic rhinitis among smokers was also reported by Desalu et al [9], but in a recent meta-analysis by Saulyte et al, there was no association between current or past tobacco use and allergic rhinitis in adults [44]. Studies have been conflicting on the association between socio-economic status (SES) and allergic rhinitis. Some studies have suggested higher prevalence of allergic rhinitis in people with high SES [9,45], while other have found not association [46].

The main limitation of this study resides in the use of screening questionnaire to diagnose asthma and allergic rhinitis. This approach is subject to recall bias, but our questionnaire was adapted from internationally validated questionnaires, and which have been used in most surveys around the world, therefore allowing our results to be comparable with those from the many other studies with similar methodological approaches [18,19]. It is possible that using spirometry (to characterise the reversibility and non-specific bronchial hyperreactivity) and allergologic tests would have refined the description of the prevalence in our study. The large sample size is a major strength of the current study, and has allowed us to derive stable estimates of disease occurrence.

## Conclusion

Prevalent asthma and allergic rhinitis in this major central African city occurred at frequencies in the lower tails of reports from around the world. A large proportion of participants with severe wheezing does not receive treatment in this setting and are therefore at risk of fatal outcome. In addition to the classical association between allergic diseases which was confirmed in our study, tobacco use and education were independent correlates of symptoms of allergic rhinitis. A nationwide survey will aid the refinement of our finding and investigate within country variations in the prevalence of asthma in Cameroon.

## Supporting Information

S1 Dataset Dataset for prevalence of asthma and rhinitis in adults in Yaounde, Cameroon. (XLS)

## References

[1] World Health Organisation. Asthma. Fact sheet n° 307, 2011. Available: http://www.who.int/mediacentre/factsheets/fs307/en/index.html.

[2] Zhang Y, Zhang L. Prevalence of allergic rhinitis in china. Allergy Asthma Immunol Res. 2014;6:105–113. 10.4168/aair.2014.6.2.105 24587945 PMC3936037

[3] Schoenwetter WF, Dupclay L Jr., Appajosyula S, Botteman MF, Pashos CL. Economic impact and quality-of-life burden of allergic rhinitis. Curr Med Res Opin. 2004;20:305–317. 15025839 10.1185/030079903125003053

[4] Bousquet J, Khaltaev N, Cruz AA, Denburg J, Fokkens WJ, Togias A, et al Allergic rhinitis and its impact on asthma (ARIA) 2008 update (in collaboration with the world health organization, ga(2)len and allergen). Allergy. 2008;63 Suppl 86:8–160. 10.1111/j.1398-9995.2007.01620.x 18331513

[5] Anandan C, Nurmatov U, van Schayck OC, Sheikh A. Is the prevalence of asthma declining? Systematic review of epidemiological studies. Allergy. 2010;65:152–167 10.1111/j.1398-9995.2009.02244.x 19912154

[6] Rutkowski K, Sowa P, Rutkowska-Talipska J, Sulkowski S, Rutkowski R. Allergic diseases: The price of civilisational progress. Postepy Dermatol Alergol. 2014;31:77–83. 10.5114/pdia.2014.40936 25097472 PMC4112251

[7] Piau JP, Massot C, Moreau D, Ait-Khaled N, Bouayad Z, Mohammad Y, et al Assessing allergic rhinitis in developing countries. Int J Tuberc Lung Dis. 2010;14:506–512. 20202311

[8] Adeloye D, Chan KY, Rudan I, Campbell H. An estimate of asthma prevalence in africa: A systematic analysis. Croat Med J. 2013;54:519–531. 24382846 10.3325/cmj.2013.54.519 PMC3893990

[9] Desalu OO, Salami AK, Iseh KR, Oluboyo PO. Prevalence of self reported allergic rhinitis and its relationship with asthma among adult nigerians. J Investig Allergol Clin Immunol. 2009;19:474–480. 20128422

[10] Nyembue TD, Jorissen M, Hellings PW, Muyunga C, Kayembe JM. Prevalence and determinants of allergic diseases in a congolese population. Int Forum Allergy Rhinol. 2012;2:285–293. 10.1002/alr.21017 22294496

[11] Erhabor GE, Agbroko SO, Bamigboye P, Awopeju OF. Prevalence of asthma symptoms among university students 15 to 35 years of age in obafemi awolowo university, ile-ife, osun state. J Asthma. 2006;43:161–164. 16517434 10.1080/02770900500499046

[12] Desalu OO, Oluboyo PO, Salami AK. The prevalence of bronchial asthma among adults in ilorin, nigeria. Afr J Med Med Sci. 2009;38:149–154. 20175418

[13] Mugusi F, Edwards R, Hayes L, Unwin N, Mbanya JC, Whiting D, et al Prevalence of wheeze and self-reported asthma and asthma care in an urban and rural area of tanzania and cameroon. Trop Doct. 2004;34:209–214. 15510944 10.1177/004947550403400408

[14] Ehrlich RI, White N, Norman R, Laubscher R, Steyn K, Lombard C, et al Wheeze, asthma diagnosis and medication use: A national adult survey in a developing country. Thorax. 2005;60:895–901. 16263947 10.1136/thx.2004.030932 PMC1747242

[15] Bureau Central des Recensements et des Etudes de Population. Rapport de présentation des résultats définitifs, 2010. Available: http://www.statistics-cameroon.org/downloads/Rapport_de_presentation_3_RGPH.pdf.

[16] Organisation Mondiale de la Santé. Les jeunes et la santé: Défi pour la société, 1986 Available: http://whqlibdoc.who.int/trs/WHO_TRS_731_fre.pdf.

[17] Bureau Central des Recensements et des Etudes de Population. Population en chiffre, 2010. Available: http://www.bucrep.cm/en/census/3rd-gphc/57-population-en-chiffre.html.

[18] Asher MI, Keil U, Anderson HR, Beasley R, Crane J, Martinez F, et al International study of asthma and allergies in childhood (ISAAC): Rationale and methods. Eur Respir J. 1995;8:483–491. 7789502 10.1183/09031936.95.08030483

[19] Burney PG, Luczynska C, Chinn S, Jarvis D. The European Community Respiratory Health Survey. Eur Respir J. 1994;7:954–960. 8050554 10.1183/09031936.94.07050954

[20] Buist AS, Vollmer WM, Sullivan SD, Weiss KB, Lee TA, Menezes AM, et al The burden of obstructive lung disease initiative (bold): Rationale and design. COPD. 2005;2:277–283. 17136954

[21] World Health Organization Obesity: Preventing and managing the global epidemic Report of WHO Consultation on Obesity, 3–5 June 1997. Geneva: WHO; 1998 10.2147/IJNRD.S78310 11234459

[22] Adams G, Gulliford MC, Ukoumunne OC, Eldridge S, Chinn S, Campbell MJ. Patterns of intra-cluster correlation from primary care research to inform study design and analysis. J Clin Epidemiol. 2004;57:785–794. 15485730 10.1016/j.jclinepi.2003.12.013

[23] Cameroon's National Institute of Statistics. Population of Cameroon: State and structure of the population Available: http://www.statistics-cameroon.org/downloads/Etat_et_structure_de_la_population.pdf.

[24] Woodward M. Epidemiology: Study design and data analysis. Chapmen & hall/crc texts in statistical science series. 2005:849.

[25] Janson C, Anto J, Burney P, Chinn S, de Marco R, Heinrich J, et al The European Community Respiratory Health Survey: What are the main results so far? European Community Respiratory Health Survey II. Eur Respir J. 2001;18:598–611. 11589359 10.1183/09031936.01.00205801

[26] Chinn S, Jarvis D, Burney P, Luczynska C, Ackermann-Liebrich U, Anto JM, et al Increase in diagnosed asthma but not in symptoms in the european community respiratory health survey. Thorax. 2004;59:646–651. 15282382 10.1136/thx.2004.021642 PMC1747094

[27] de Marco R, Cappa V, Accordini S, Rava M, Antonicelli L, Bortolami O, et al Trends in the prevalence of asthma and allergic rhinitis in italy between 1991 and 2010. Eur Respir J. 2012;39:883–892. 10.1183/09031936.00061611 22005911

[28] Zhu H, Yao WZ, Shen N, Zhang LQ, Wang XH, Liang YJ, et al [Epidemiological survey of asthma in a rural area of yanqing county in beijing]. Beijing Da Xue Xue Bao. 2007;39:494–497. 17940567

[29] Song WJ, Kang MG, Chang YS, Cho SH. Epidemiology of adult asthma in asia: Toward a better understanding. Asia Pac Allergy. 2014;4:75–85. 10.5415/apallergy.2014.4.2.75 24809012 PMC4005350

[30] Amberbir A, Medhin G, Hanlon C, Britton J, Venn A, Davey G. Frequent use of paracetamol and risk of allergic disease among women in an ethiopian population. PLoS One. 2011;6:e22551 10.1371/journal.pone.0022551 21811632 PMC3141069

[31] Asher MI, Montefort S, Bjorksten B, Lai CK, Strachan DP, Weiland SK, et al Worldwide time trends in the prevalence of symptoms of asthma, allergic rhinoconjunctivitis, and eczema in childhood: Isaac phases one and three repeat multicountry cross-sectional surveys. Lancet. 2006;368:733–743. 16935684 10.1016/S0140-6736(06)69283-0

[32] Liu AH. Hygiene theory and allergy and asthma prevention. Paediatr Perinat Epidemiol. 2007;21 Suppl 3:2–7. 17935569 10.1111/j.1365-3016.2007.00878.x

[33] Custovic A, Simpson A, Woodcock A. Importance of indoor allergens in the induction of allergy and elicitation of allergic disease. Allergy. 1998;53:115–120. 10096822 10.1111/j.1398-9995.1998.tb05011.x

[34] El Fekih L, Mjid M, Souissi Z, Ben Hmida A, El Gueddari Y, Douagui H, et al Étude de la sensibilisation aux 3 acariens (dermatophagoïdes pteronyssinus, dermatophagoïdes farinae, blomia tropicalis) au maghreb et en afrique subsaharienne dans une population de patients consultant pour une rhinite et/ou un asthme. Revue Française d'Allergologie. 2014;54:107–112.

[35] Pefura-Yone EW, Kengne AP, Kuaban C. Sensitisation to mites in a group of patients with asthma in yaounde, cameroon: A cross-sectional study. BMJ open. 2014;4:e004062 10.1136/bmjopen-2013-004062 24390384 PMC3902465

[36] Pefura-Yone EW, Mbele-Onana CL, Balkissou AD, Kenmegne-Noumsi EC, Boulleys-Nana JR, Kolontchang-Yomi BL, et al. Perennial aeroallergens sensitisation and risk of asthma in african children and adolescents: A case-control study. J Asthma. 2014:1–5. 10.3109/02770903.2014.995306 25494554

[37] Canova C, Heinrich J, Anto JM, Leynaert B, Smith M, Kuenzli N, et al The influence of sensitisation to pollens and moulds on seasonal variations in asthma attacks. Eur Respir J. 2013;42:935–945. 10.1183/09031936.00097412 23471350 PMC3787817

[38] Musafiri S, van Meerbeeck J, Musango L, Brusselle G, Joos G, Seminega B, et al Prevalence of atopy, asthma and copd in an urban and a rural area of an african country. Respir Med. 2011;105:1596–1605. 10.1016/j.rmed.2011.06.013 21783353

[39] Arif AA, Delclos GL, Lee ES, Tortolero SR, Whitehead LW. Prevalence and risk factors of asthma and wheezing among us adults: An analysis of the nhanes iii data. Eur Respir J. 2003;21:827–833. 12765429 10.1183/09031936.03.00054103a

[40] Ozdoganoglu T, Songu M. The burden of allergic rhinitis and asthma. Ther Adv Respir Dis. 2012;6:11–23. 10.1177/1753465811431975 22179899

[41] Sonomjamts M, Dashdemberel S, Logii N, Nakae K, Chigusa Y, Ohhira S, et al Prevalence of asthma and allergic rhinitis among adult population in ulaanbaatar, mongolia. Asia Pac Allergy. 2014;4:25–31. 10.5415/apallergy.2014.4.1.25 24527407 PMC3921862

[42] Bauchau V, Durham SR. Prevalence and rate of diagnosis of allergic rhinitis in europe. Eur Respir J. 2004;24:758–764. 15516669 10.1183/09031936.04.00013904

[43] Meltzer EO, Blaiss MS, Naclerio RM, Stoloff SW, Derebery MJ, Nelson HS, et al Burden of allergic rhinitis: Allergies in America, Latin America, and Asia-Pacific adult surveys. Allergy Asthma Proc. 2012;33 Suppl 1:S113–141. 10.2500/aap.2012.33.3603 22981425

[44] Saulyte J, Regueira C, Montes-Martinez A, Khudyakov P, Takkouche B. Active or passive exposure to tobacco smoking and allergic rhinitis, allergic dermatitis, and food allergy in adults and children: A systematic review and meta-analysis. PLoS Med. 2014;11:e1001611 10.1371/journal.pmed.1001611 24618794 PMC3949681

[45] Braback L, Hjern A, Rasmussen F. Social class in asthma and allergic rhinitis: A national cohort study over three decades. Eur Respir J. 2005;26:1064–1068. 16319336 10.1183/09031936.05.00022105

[46] Leynaert B, Neukirch C, Liard R, Bousquet J, Neukirch F. Quality of life in allergic rhinitis and asthma. A population-based study of young adults. Am J Respir Crit Care Med. 2000;162:1391–1396. 11029350 10.1164/ajrccm.162.4.9912033

